# Micro environment data on the Daeungbojeon hall and the Palsangjeon hall of the Beopjusa temple in Republic of Korea

**DOI:** 10.1016/j.dib.2021.107515

**Published:** 2021-10-27

**Authors:** BoA Lim, YoungHee Kim, JiHee Park, JeungMin Lee

**Affiliations:** Restoration Technology Division, National Research Institute of Cultural Heritage, 132 Munji-ro, Yuseong-gu, Daejeon 34122, Republic of Korea

**Keywords:** Micro environment, Wooden building, Long-term monitoring, Monthly average, Time average value

## Abstract

This article provides long-term environmental change data for wooden buildings; it also reflects environmental data provided by the Korea Meteorological Administration. In the case of field survey, data logger was installed on the left rear and right front sides of the buildings. Datasets on the Beopjusa temple were collected at 1 h intervals in each building. Korea Meteorological Administration data was collected from public database(data.kma.go.kr) and all data processed in excel. The data was collected at two sites from Daeungbojeon hall and Palsangjeon hall in the Beopjusa temple, Republic of Korea. Data sets at 1 h intervals are provided by collecting more than 170,000 pieces of data for each building. And monthly average dataset and difference value of time average data between inside and outside are provided. This data can be used as basic data for environmental change researcher or simulation researcher of wood condition.

## Specifications Table

**Table:** 

Subject	Environmental Science
Specific subject area	Climatology, Micro environment, Difference value between inside and outside of wooden building
Type of data	Table Graph
How data were acquired	Raw data: In the case of field survey, data loggers were installed on the left rear and right front sides of the buildings. Datasets on the Beopjusa temple were collected at 1 h intervals in each buildings. Korea Meteorological Administration data was collected from public database(data. kma.go.kr) and all data processed in excel. Analysed data: raw data was used to calculate with monthly average data and time average value inside wooden building.
Data format	Raw Analysed
Parameters for data collection	Air temperature and relative humidity data, Monthly average and time average data, Difference between inside and outside, The datasets collected from June 1, 2015 to April 30, 2020.
Description of data collection	The hourly data provided by the Korea Meteorological Administration database was used, and we collected on the data 1 h intervals that are automatically saved inside Daeungbojeon hall and Palsangjeon hall in Beopjusa temple.
Data source location	Institution: Beopjusa temple and Boeun weather observatory City: Boeun Country: the Republic of Korea Daeungbojeon hall in Beopjusa temple; 36° 54′ 29.30″, 127° 83′ 35.29″, Palsangjeon hall in Beopjusa temple; 36° 54′ 29.30″, 127° 83′ 83.40″ Boeun weather observatory; 36° 48′ 76.10″, 127° 73′ 41.50″ Primary data sources: hourly data on collecting from June 1, 2015 to April 30, 2020 in Beopjusa temple and Boeun observatory data from public database(data.kma.go.kr).
Data accessibility	Repository name: Mendeley Data Data identification number: DOI: 10.17632/mwgjgbb84z.4 Direct URL to data: http://data.mendeley.com/datasets/mwgjgbb84z/4

## Value of the Data

• This is the first time that real-time temperature and relative humidity have been collected over a long period of time using the logger devices in wooden architectural heritage.
• The data provided in this article can be used for comparing the differences between its weather observatory and wooden architectural heritage by the trends of temperature and relative humidity.
• This data can be interesting to researchers carrying out studies on the environment of wooden buildings, and can provide long-term indoor/outdoor comparative data.
• This data is also useful for studying the advantages and disadvantages of wooden buildings in response to climate change. The wooden building has the advantage of maintaining a constant indoor temperature and humidity despite changes in air temperature and relative humidity. On the other hand, if dew condensation occurs due to temperature change, the damage may be accelerated compared to other materials.
• This data is also useful for those doing climate simulations.

## 1 Data Description

**Fig. 1:**
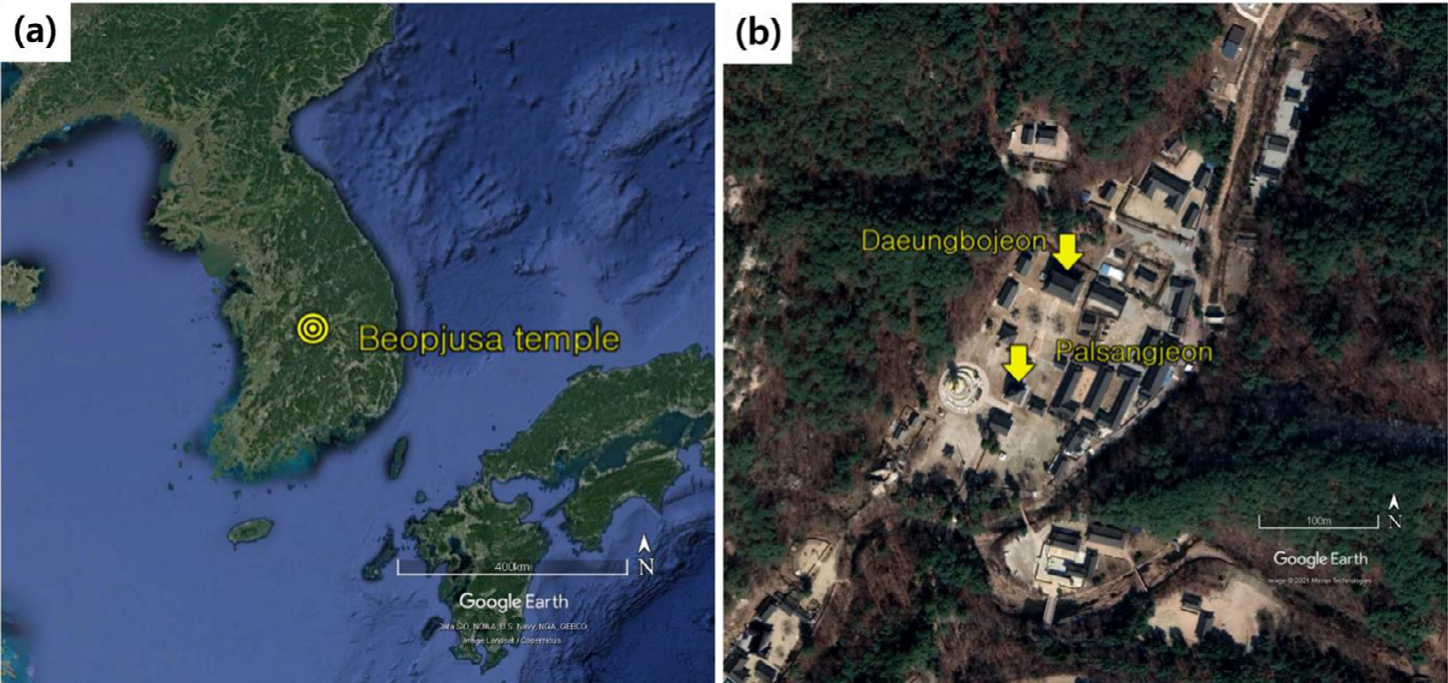
Location of Beopjusa temple (a) and Buildings (b) on Google Maps [5].

Beopjusa temple is located in Boeun city, the center of South Korea and has wooden structures designated as a National Treasure, Palsangjeon hall and Treasure, Daeungbojeon hall (Fig. 1) [1].

**Fig. 2:**
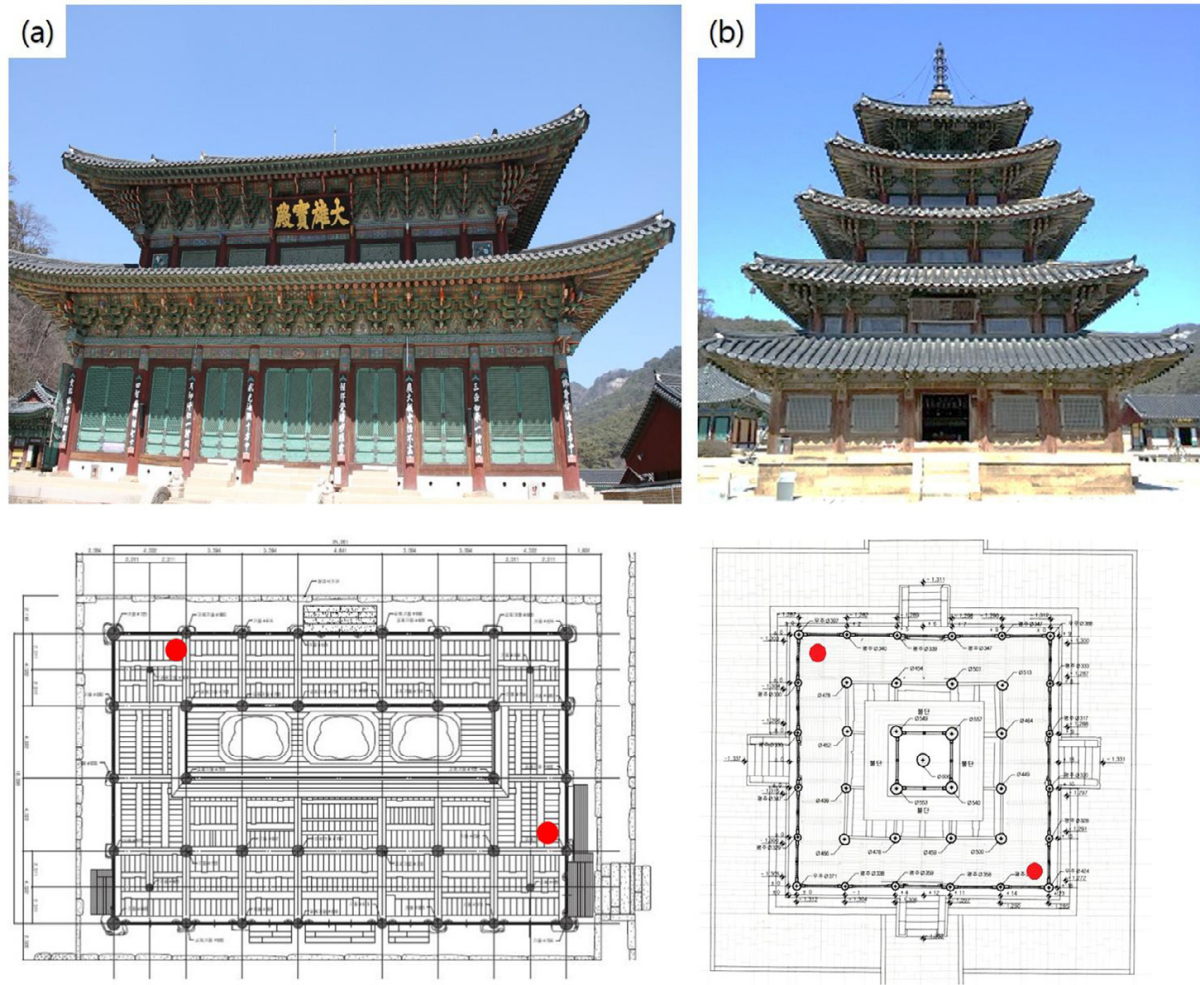
Front view of Daeungbojeon hall (a) and Palsangjeon hall (b) in the Beopjusa temple. The red dots are the installation location on the floor plan of Daeungbojeon and Palsangjeon buildings.

Fig. 2 is a front photo of the Daeungbojeon hall and the Palsangjeon hall at Beopjusa temple in Boeun, and you can see the scale of the building. The Daeungbojeon hall is a two-story wooden building with a height of about 19m and the height of the lower floors is significantly higher than the upper floors(Fig. 2 (a)). The Palsangjeon hall is a 5-story wooden building measuring about 21m and is the tallest building among wooden architectural heritage (Fig. 2 (b)). Both buildings are included in the large buildings of wooden architecture cultural heritage in South Korea.

**Fig. 3:**
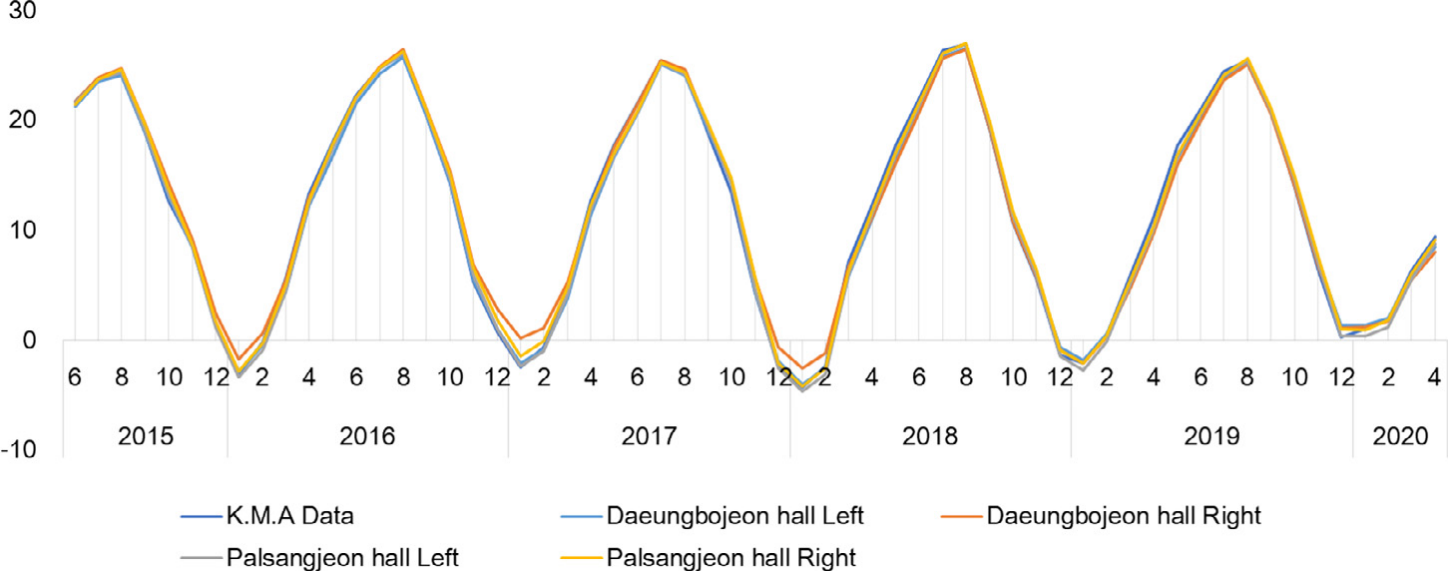
The trends of temperature(°C) in monthly average value between Korea Meteorological Administration(Boeun site) value, Daeungbojeon hall and Palsangjeon hall.

**Fig. 4:**
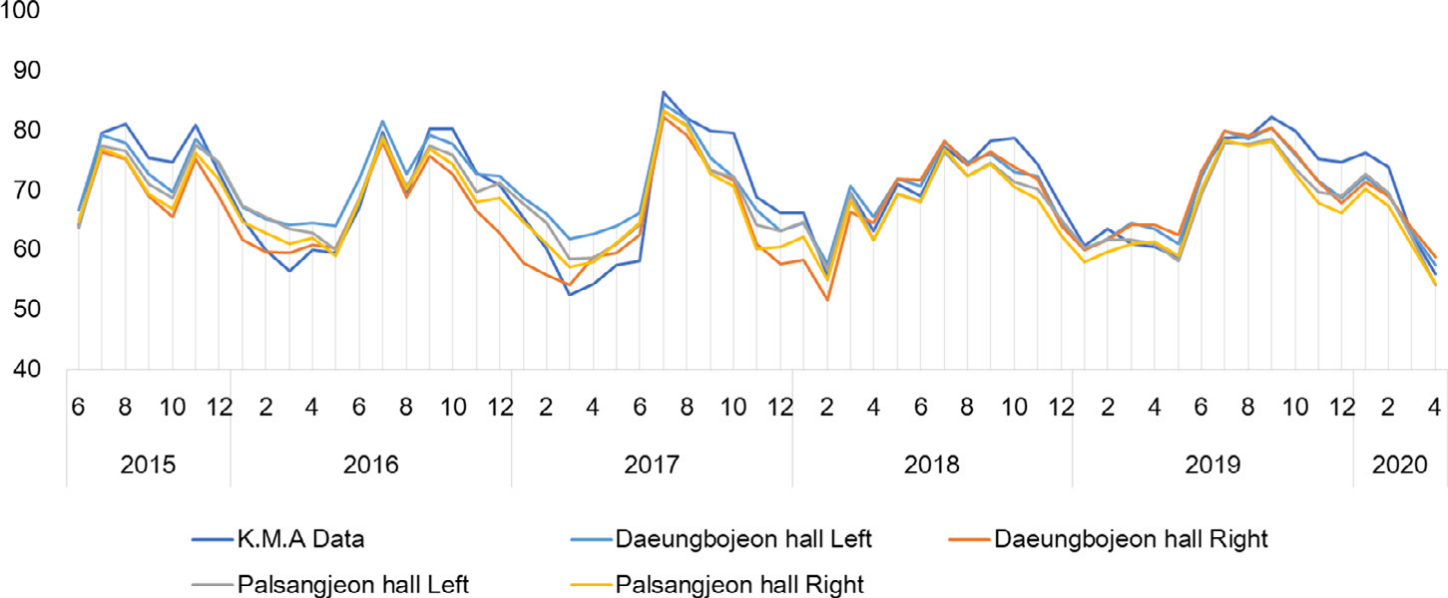
The trends of relative humidity(%) in monthly average value between Korea Meteorological Administration(Boeun site) value, Daeungbojeon hall and Palsangjeon hall.

**Fig. 5:**
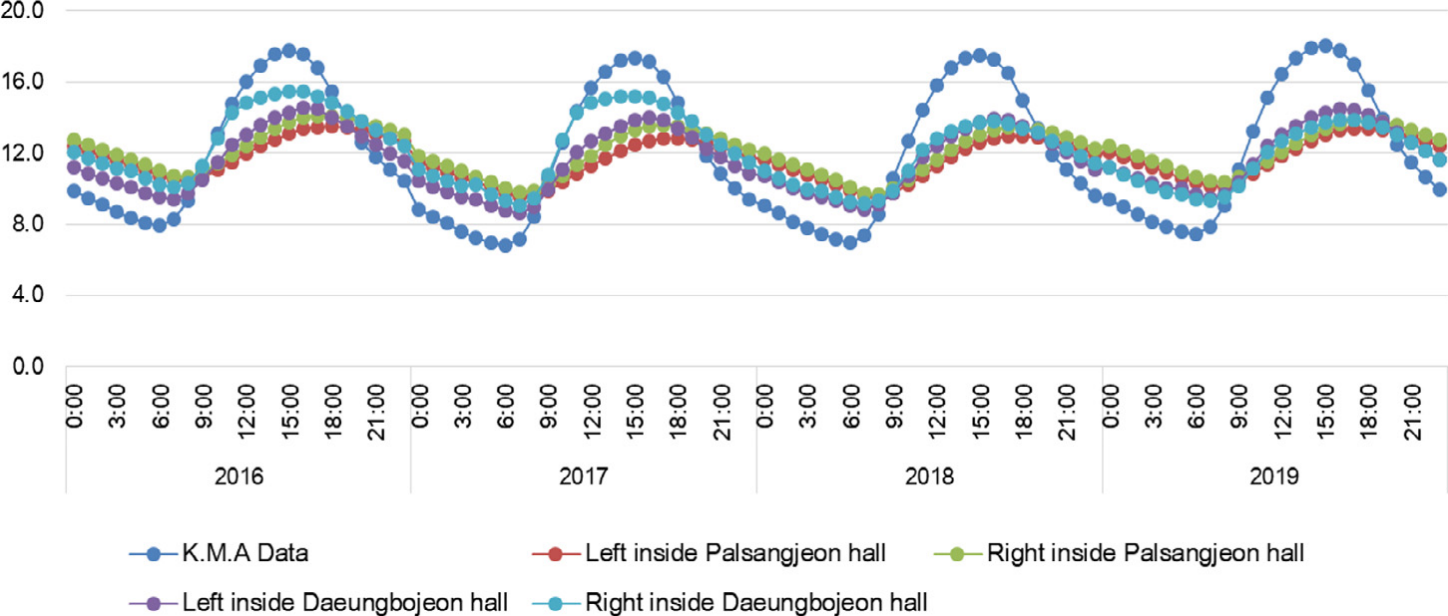
The trends of temperature(°C) in time average value between Korea Meteorological Administration(Boeun site) value, Daeungbojeon hall and Palsangjeon hall.

**Fig. 6:**
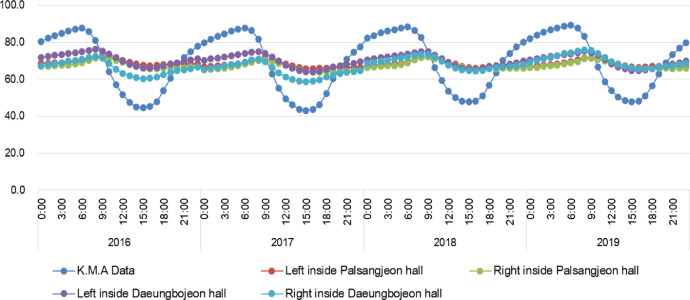
The trends of relative humidity(%) in time average value between Korea Meteorological Administration(Boeun site) value, Daeungbojeon hall and Palsangjeon hall.

We provide real time datasets collected from June 1, 2015 to April 30, 2020 on the Daeungbojeon hall and Palsangjeon hall in Beopjusa temple, Boeun, the Republic of Korea. This data parameters measured include air temperature and relative humidity. All parameters were measured inside two wooden buildings using US12-011 data loggers with temperature and relative humidity sensors [2]. A total of 172,416 measurements of each parameter were recorded from June 1, 2015 to April 30, 2020. 43,104 data per parameter were collected at one site inside one wooden building. This article contains the raw data, provided by the Korea Meteorological Administration, on the real time temperature and relative humidity of Boeun observatory [3]. Table 1 shows the total number of temperature and relative humidity data obtained by each building and year. This dataset including raw data of Korea Meteorological Administration is available in Mendeley Data Repository(DOI: 10.17632/mwgjgbb84z.4). These data was used to calculation of monthly average and time average value. And graphs of monthly average and time average data are presented in this article (Fig. 3, Fig. 4, Fig. 5, Fig. 6). Table 2 shows the annual mean, maximum and minimum values of temperature and relative humidity in each building.

**Table 1:** Total number of measurement data of each parameter and location.

Parameter	Temperature (°C)					Humidity (%)				
Location	Daeungbojeon		Palsangjeon			Daeungbojeon		Palsangjeon		
Year	Left	Right	Left	Right	K.M.A Boeun	Left	Right	Left	Right	K.M.A Boeun
2015	5136	5136	5136	5136	5134	5136	5136	5136	5136	5134
2016	8784	8784	8784	8784	8784	8784	8784	8784	8784	8784
2017	8760	8760	8760	8760	8758	8760	8760	8760	8760	8758
2018	8760	8760	8760	8760	8760	8760	8760	8760	8760	8760
2019	8760	8760	8760	8760	8756	8760	8760	8760	8760	8756
2020	2904	2904	2904	2904	2902	2904	2904	2904	2904	2902

Total	43104	43104	43104	43104	43094	43104	43104	43104	43104	43094

**Table 2:** The annual mean, maximum and minimum values of temperature and relative humidity.

		Temperature (°C)				Relative humidity (%)			
Location	Statistics	2016	2017	2018	2019	2016	2017	2018	2019
Korea Meteorological Administration (Boeun)	Mean	12.3	11.5	11.7	12.1	71.1	69.4	69.9	69.8
	Max	26.0	25.5	26.9	25.5	81.5	84.4	78.0	80.4
	Min	−3.1	−2.5	−4.3	−2.1	64.0	61.8	57.6	59.9

Daeungbojeon hall Left	Mean	11.9	11.2	11.3	11.9	66.2	64.4	68.5	70.0
	Max	25.7	25.1	26.6	25.2	78.0	82.1	78.2	80.4
	Min	−2.9	−2.1	−4.0	−1.8	59.4	54.1	51.6	60.0

Daeungbojeon hall Right	Mean	12.9	12.2	11.5	11.6	68.4	67.6	70.3	70.5
	Max	26.5	25.5	26.5	25.1	80.1	86.3	78.6	82.1
	Min	−1.7	−0.6	−2.6	−2.1	56.4	52.5	55.7	58.8

Palsangjeon hall Left	Mean	12.1	11.3	11.4	11.8	69.2	67.6	68.3	68.2
	Max	26.1	25.2	26.9	25.4	79.0	83.2	76.4	78.5
	Min	−3.4	−2.6	−4.7	−2.7	59.9	58.4	56.7	58.2

Palsangjeon hall Right	Mean	12.5	11.8	11.7	12.2	67.9	66.2	67.4	67.5
	Max	26.3	25.3	27.0	25.6	78.7	83.2	76.8	78.4
	Min	−2.8	−2.1	−4.2	−2.1	58.9	57.2	54.9	57.9

Wooden buildings, a traditional Korean building, provide a relatively stable environment from the surrounding environment by the characteristics of wood [4]. We intend to collecting environmental data of wooden buildings and provide long-term monitoring data inside wooden buildings. Our datasets provide information about the micro environment of wooden buildings.

## 2 Experimental Design, Materials and Methods

### 2.1 Survey area

The study area is Beopjusa temple, Boeun-city, Chungcheongbuk-do, the center of South Korea; it is between latitude 36.52°-36.56°and longitude 127.68°-127.84°. The topography is valley and elevation averaged about 350 m above mean sea level. The temple buildings are oriented to the southwest. The climate of region is characterized by high temperatures in summer and high rainfall, and low temperatures and dry climates in winter.

Data were collected from field survey and public database(data.kma.go.kr). For field survey, it was measured at targeted two buildings, Daeungbojeon hall and Palsangjeon hall from the Beopjusa temple(Boeun-eup, Boeun-gun, Chungcheongbuk-do, Republic of Korea).

### 2.2 Data acquisition

This data was automatically measured every hour on the hour. The data collection period is from June 1, 2015 to April 30, 2020. We also applied the same period of time for the public database (data.kma.go.kr). HOBO Temperature/Relative Humidity data loggers(U12-011, Onset, USA) were installed on the left rear and right front sides of the two buildings, respectively (Fig. 2). The installation location is appropriately placed at the top of the first floor. The instrumental characteristics of data logger, US12-011 are given in Table 3.

**Table 3:** Instrumental characteristic of data logger(US12-011).

Specification	Temperature (°C)	Relative Humidity (%)
Measurement range	−20° to 70°C (−4° to 158°F)	5% to 95% RH

Accuracy	± 0.35°C from 0° to 50°C (± 0.63°F from 32° to 122°F)	±2.5% from 10% to 90% RH (typical), to a maximum of ±3.5% including hysteresis

Resolution	0.03°C at 25°C (0.05°F at 77°F)	0.03% RH

Drift	0.1°C/year (0.2°F/year)	<1% per year typical

Response time in airflow of 1 m/s (2.2 mph)	6 min, typical to 90%	1 min, typical to 90%

Sample Rate	1 s to 18 h, user selectable	
Time accuracy	± 1 min per month at 25°C (77°F)	

### 2.3 Data processing

The Excel 2016 program was used to convert the collected data into monthly average and time average data. We calculated the monthly average and time average value using the pivot table function of Excel program and confirmed the trends of temperature and relative humidity between the inside of wooden buildings and outside data of public database. The time average data was calculated using data with all data for 12 months from 2016 to 2019.

## Ethics Statement

This data is NOT relevant for human project and animal studies.

## CRediT authorship contribution statement

**BoA Lim:** Data curation, Investigation, Visualization, Writing – original draft. **YoungHee Kim:** Investigation, Supervision, Writing – review & editing. **JiHee Park:** Project administration. **JeungMin Lee:** Investigation.

## Declaration of Competing Interest

The authors declare that they have no known competing financial interests or personal relationships which have or could be perceived to have influenced the work reported in this article.
